# Dietary Mg^2+^ Intake and the Na^+^/Mg^2+^ Exchanger SLC41A1 Influence Components of Mitochondrial Energetics in Murine Cardiomyocytes

**DOI:** 10.3390/ijms21218221

**Published:** 2020-11-03

**Authors:** Zuzana Tatarkova, Jeroen H. F. de Baaij, Marian Grendar, Jörg R. Aschenbach, Peter Racay, Caro Bos, Gerhard Sponder, Joost G. J. Hoenderop, Monika Röntgen, Monika Turcanova Koprusakova, Martin Kolisek

**Affiliations:** 1 Department of Medical Biochemistry, Jessenius Faculty of Medicine, Comenius University in Bratislava, Mala Hora 4D, 036 01 Martin, Slovakia; zuzana.tatarkova@uniba.sk (Z.T.); peter.racay@uniba.sk (P.R.); 2 Department of Physiology, Radboud Institute for Molecular Life Sciences, Radboud University Medical Center, 6500HB Nijmegen, The Netherlands; Jeroen.deBaaij@radboudumc.nl (J.H.F.d.B.); Caro.Bos@radboudumc.nl (C.B.); Joost.Hoenderop@radboudumc.nl (J.G.J.H.); 3 Biomedical Center Martin, Jessenius Faculty of Medicine, Comenius University in Bratislava, Mala Hora 4D, 036 01 Martin, Slovakia; marian.grendar@uniba.sk; 4 Institute of Veterinary Physiology, Freie Universität Berlin, Oertzenweg 19b, 14163 Berlin, Germany; joerg.aschenbach@fu-berlin.de (J.R.A.); gerhard.sponder@fu-berlin.de (G.S.); 5 Leibniz Institute for Farm Animal Biology, Wilhelm-Stahl-Allee 2, 18196 Dummerstorf, Germany; roentgen@fbn-dummerstorf.de; 6 Clinic of Neurology, University Hospital in Martin, Kollarova 4248/2, 036 01 Martin, Slovakia; koprusakova@gmail.com

**Keywords:** magnesium, cardiomyocyte, Krebs cycle, electron transport chain, oxidative phosphorylation, Na^+^/Mg^2+^ exchanger

## Abstract

Cardiomyocytes are among the most energy-intensive cell types. Interplay between the components of cellular magnesium (Mg) homeostasis and energy metabolism in cardiomyocytes is poorly understood. We have investigated the effects of dietary Mg content and presence/functionality of the Na^+^/Mg^2+^ exchanger SLC41A1 on enzymatic functions of selected constituents of the Krebs cycle and complexes of the electron transport chain (ETC). The activities of aconitate hydratase (ACON), isocitrate dehydrogenase (ICDH), α-ketoglutarate dehydrogenase (KGDH), and ETC complexes CI–CV have been determined in vitro in mitochondria isolated from hearts of wild-type (WT) and *Slc41a1*^−/−^ mice fed a diet with either normal or low Mg content. Our data demonstrate that both, the type of Mg diet and the *Slc41a1* genotype largely impact on the activities of enzymes of the Krebs cycle and ETC. Moreover, a compensatory effect of *Slc41a1*^−/−^ genotype on the effect of low Mg diet on activities of the tested Krebs cycle enzymes has been identified. A machine-learning analysis identified activities of ICDH, CI, CIV, and CV as common predictors of the type of Mg diet and of CII as suitable predictor of *Slc41a1* genotype. Thus, our data delineate the effect of dietary Mg content and of SLC41A1 functionality on the energy-production in cardiac mitochondria.

## 1. Introduction

Cardiovascular diseases (CVD) account for ~31% of all estimated deaths worldwide (www.who.int). Ageing *per se* is the major intrinsic risk factor for heart failure and CVD [1]. An unhealthy diet including excessive food intake, alcohol consumption, smoking, low physical activity, and stress have been identified as the most prominent among known extrinsic risk factors for CVD. At the cellular level, all the aforementioned external risk factors have been associated with premature cardiac ageing [1,2,3,4] and modulation/deterioration of mitochondrial energetics in cardiomyocytes [5,6,7].

The typical Western diet, which is rich in processed food with high sugar and fat content, lacks sufficient amounts of Mg [8,9]. This can lead to subclinical intracellular Mg deficiency, which is rarely diagnosed [9,10]. In particular, chronic Mg deficiency imposes a serious problem, leading to health complications including CVD [9,11].

Magnesium plays a plethora of essential roles in securing the normal physiology of cardiomyocytes, including the regulation of Ca^2+^ and K^+^ channels [12,13,14]. It is also essential for energy production in the mitochondria of cardiomyocytes and ATP stabilization [10,15]. Intracellular Mg deficiency results either from insufficient Mg^2+^ influx or excessive Mg^2+^ efflux, or a combination of both [10,16]. Growing evidence indicates that the disintegration of intracellular Mg homeostasis negatively affects cellular energetics, mitochondrial performance, and overall mitochondrial homeostasis [17,18,19,20,21].

SLC41A1 (solute carrier family 41 member A1) is presently the only known eukaryotic Mg^2+^ efflux system that is integral to the cytoplasmic membrane and that is ubiquitously expressed in various tissues and cell types [21,22,23,24]. However, the greatest expression of *Slc41a1* has been detected in heart, followed by pancreas and skeletal muscle (www.proteinatlas.org). Together with TRPM7 (transient receptor potential melastatin type 7), it forms the major Mg^2+^ transport circuit in the cytoplasmic membrane [21,22,23]. The *modus operandi* of SLC41A1 has been researched in HEK293 (human embryonic kidney) cells with inducible overexpression of the carrier, and SLC41A1 has been demonstrated to operate as an Na^+^/Mg^2+^ exchanger (NME) [22,23]. Mandt and colleagues concluded that the Mg^2+^ transport function of SLC41A1 is regulated primarily through an endosomal recycling mechanism involving the N-terminal cytoplasmic domain of the transporter [25]. The regulatory modalities of SLC41A1 include: cAMP-dependent PKA (enhances NME activity of SLC41A1) [23,26], Akt/PKB (inhibits NME activity of SLC41A1) [17,27], insulin (inhibits NME activity of SLC41A1 via IR–PI3K–PDK1–Akt/PKB signaling cascade) [17,27], and possibly also neuritin, growth hormone, leptin, EGF, PDGF, IGF-1, and extracellular polyvalent ligands [27].

Heart tissues are energy- and Mg-demanding: the human heart consumes ~10% of whole-body fuel consumption because of its high metabolic activity [11,28,29]. It is able to produce energy from fatty acids, but also from several other substrates including glucose, pyruvate, and lactate [30]. Thus the β-oxidation, the Krebs cycle (also known as the tricarboxylic acid (TCA) cycle) and the ETC are essential for energy production in the heart.

The Krebs cycle harnesses the chemical energy of acetyl-CoA, and through the generation of the reduced form of electron carriers, NADH and FADH_2_, makes it available for ETC and the associated increase in ATP. Magnesium is an important coherent controller of glycolysis and the Krebs cycle [31]. It has been shown to enhance the activity of three principal mitochondrial dehydrogenases involved in energy metabolism, namely pyruvate dehydrogenase (PDH), ICDH, and KGDH [32]. Whereas ICDH and KGDH are stimulated directly by the Mg^2+^-isocitrate complex and free ionized Mg^2+^, respectively, the activator effect of Mg^2+^ on PDH is indirect through pyruvate dehydrogenase phosphatase [33,34,35]. Mg^2+^ also plays an important role in the modulation of F_1_F_o_-ATPase (CV) activity and stabilizes ATP in the intracellular milieu [36,37,38]. The metabolic activity of mitochondria depends on a sufficient supply of Mg^2+^ from the cytoplasm [11,20,39,40]. Given that SLC41A1 activity regulates the intracellular Mg^2+^ concentration ([Mg^2+^]_i_) [17,22,23,24,25,26], SLC41A1 can be expected to interfere with the control of mitochondrial energy metabolism.

In this work, we have examined whether the presence or absence of functional SLC41A1, influences the enzymatic activities of the key components of the Krebs cycle (ACON, ICDH, and KGDH) and/or activities of the complexes of ETC (CI–CIV) including F_1_F_o_-ATPase (CV) in mitochondria of cells from murine hearts. Furthermore, the impact of a normal Mg diet (NMgD) and of a low Mg diet (LMgD) on the activities of the aforementioned components of the Krebs cycle and ETC in cardiac mitochondria isolated from hearts of knock-out (KO; *Slc41a1*^−/−^) and wild-type (WT; *Slc41a1*^+/+^) mice have been evaluated.

## 2. Results

### 2.1. Effect of Dietary Mg Content and Slc41a1 Genotype on Activities of ACON, ICDH, and KGDH in Lysates of Mitochondria of Murine Cardiac Cells

**Figure 1:**
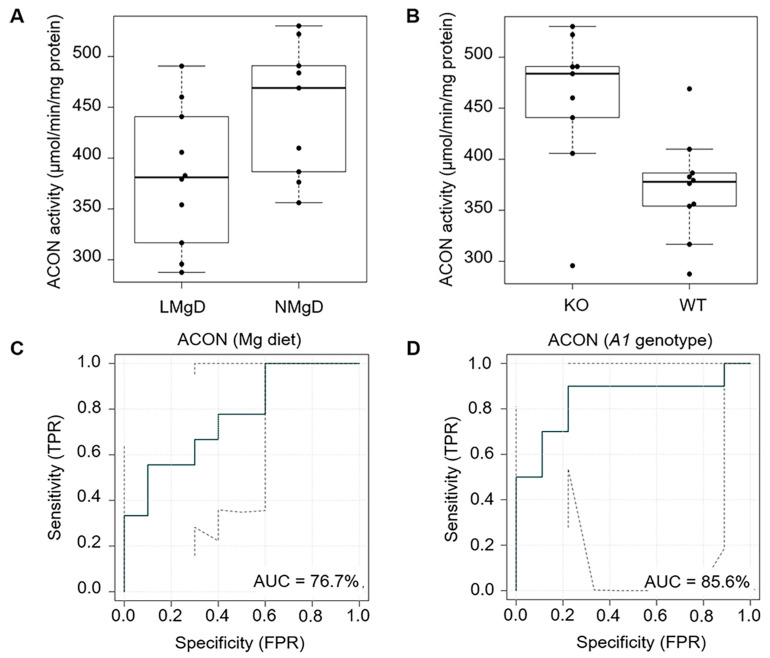
Impact of dietary Mg content and of *Slc41a1* genotype on the activity of aconitase (ACON) in murine cardiac mitochondria. (**A**) Box-and-whisker plot overlaid with dot plot showing the influence of dietary Mg content (LMgD vs. NMgD) and (**B**) of *Slc41a1* genotype (*Slc41a1*^−/−^ vs. *Slc41a1*^+/+^) on ACON activities in murine mitochondria. For both, A and B, the original, source data and corresponding medians are indicated for each group. (**C**,**D**) Empirical ROC (receiver operating characteristic) curves with the area under the ROC curve (AUC) for (**C**) the type of Mg diet as predictor of ACON activity and (**D**) the *Slc41a1* genotype as predictor of ACON activity. Abbreviations: *A1*, *Slc41a1*; FPR, false-positive rate; KO, knock-out; TPR, true-positive rate; WT, wild-type.

First, we examined the impact of LMgD on the activity of ACON in lysates of murine *Slc41a1*^+/+^ and *Slc41a1*^−/−^ heart mitochondria. ACON is an enzyme that catalyzes the reversible 2-step (de)hydration conversion between citrate as a substrate and the final product isocitrate via the intermediate, *cis*-aconitate [41]. Two-factorial ANOVA (2F-ANOVA) indicated that low dietary Mg decreased ACON activity (*p* < 0.01); whereas absence of the *Slc41a1* gene (*Slc41a1*^−/−^ cf. *Slc41a1*^+/+^ animals) increased ACON activity (*p* < 0.01) in heart cell mitochondria (Figure 1A,B). There was no statistically significant interaction (*p* = 0.50), indicating that both factors superimposed on each other independently. The eROC (receiver operating characteristic) curve analyses revealed 76.7% and 85.6% ability to discriminate ACON activities based on the type of Mg diet and *Slc41a1* genotype, respectively (Figure 1C,D); the uncertainty of the ROC curve estimate was considered, as shown in Figure 1C,D.

**Figure 2:**
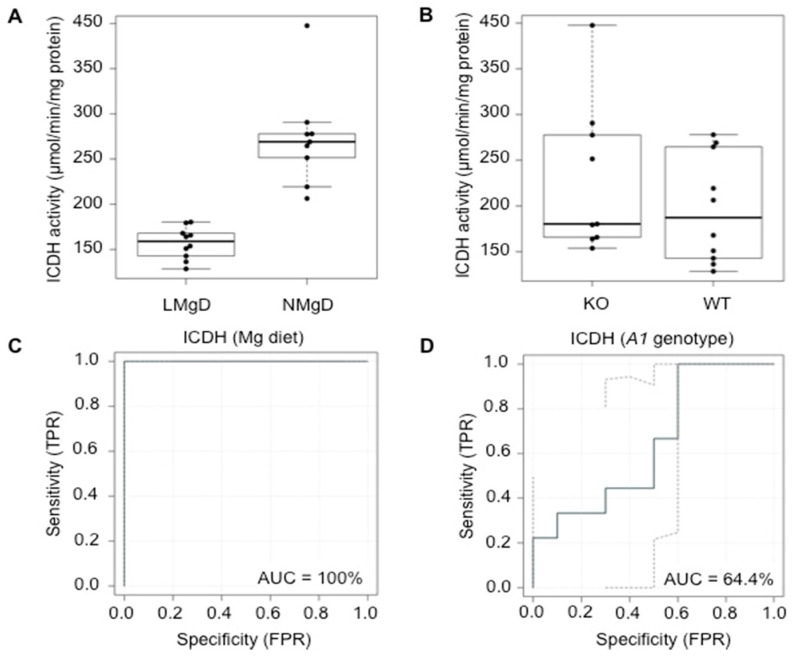
Impact of dietary Mg content and of *Slc41a1* genotype on activity of isocitrate dehydrogenase (ICDH) in murine cardiac mitochondria. (**A**) Box-and-whisker plot overlaid with dot plot showing the influence of dietary Mg content (LMgD vs. NMgD) and (**B**) *Slc41a1* genotype (*Slc41a1*^−/−^ vs. *Slc41a1*^+/+^) on ICDH activities in murine mitochondria. For both, A and B, the original source data and corresponding medians are indicated for each group. (**C**,**D**) Empirical ROC (receiver operating characteristic) curves with the area under the ROC curve (AUC) for (**C**) the type of Mg diet as predictor of ICDH activity and (**D**) the *Slc41a1* genotype as predictor of ICDH activity. Abbreviations: *A1*, *Slc41a1*; FPR, false-positive rate; KO, knock-out; TPR, true-positive rate; WT, wild-type.

Next, we examined the effect of LMgD on the activity of mitochondrial NAD^+^-dependent ICDH, an enzyme that catalyzes the regulated conversion (oxidative decarboxylation) of isocitrate to α-ketoglutarate and CO_2_. This is the first of four oxidative steps within the Krebs cycle and is the key rate-limiting step of this cycle [42]. The results were qualitatively comparable to those obtained for ACON. 2F-ANOVA revealed a significant decrease (*p* < 0.001) of ICDH activity in cardiac mitochondria of animals fed LMgD compared with animals fed NMgD; whereas the *Slc41*^−/−^ genotype tended to increase the activity of ICDH in cardiac mitochondria (*p* = 0.059; Figure 2A,B). Again, no significant interaction (*p* = 0.31) was detected between dietary Mg content and *Slc41a1* genotype, indicating that the type of response for one factor was not dependent on the type of response for the other factor. The utilization of eROC curve analyses revealed 100% and 64.4% ability to discriminate ICDH activities based on the type of Mg diet and on *Slc41a1* genotype, respectively (Figure 2C,D); the uncertainty of the ROC curve estimate was considered (Figure 2C,D).

**Figure 3:**
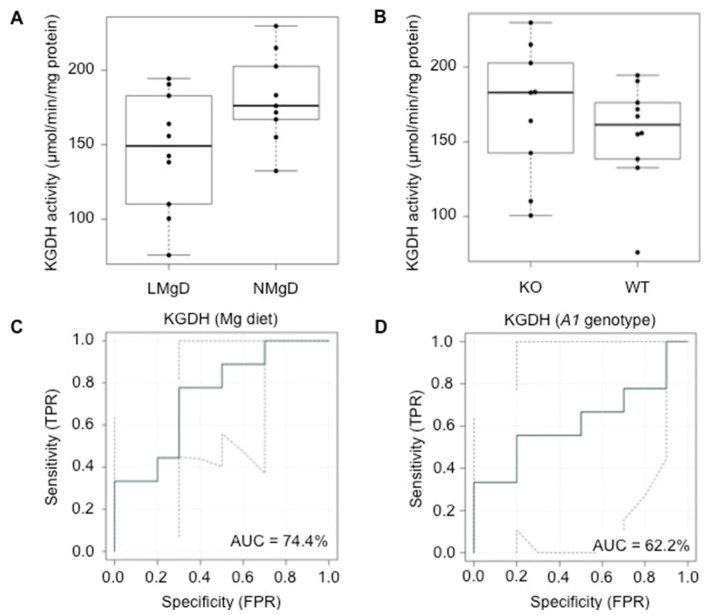
Impact of dietary Mg content and *Slc41a1* genotype on the activity of α-ketoglutarate dehydrogenase (KGDH) in murine cardiac mitochondria. (**A**) Box-and-whisker plot overlaid with dot plot showing the influence of dietary Mg content (LMgD vs. NMgD) and (**B**) *Slc41a1* genotype (*Slc41a1*^−/−^ vs. *Slc41a1*^+/+^) on KGDH activities in murine mitochondria. For both, A and B, the original source data and corresponding medians are indicated for each group. (**C**,**D**) Empirical ROC (receiver operating characteristic) curves with the area under the ROC curve (AUC) for (**C**) the type of Mg diet as predictor of KGDH activity and (**D**) the *Slc41a1* genotype as predictor of KGDH activity. Abbreviations: *A1*, *Slc41a1*; FPR, false-positive rate; KO, knock-out; TPR, true-positive rate; WT, wild-type.

KGDH mediates the tightly regulated conversion of α-ketoglutarate to succinyl-CoA while reducing NAD^+^ to NADH and thereby supplying electrons for the respiratory chain. It represents a key control point in the Krebs cycle. It is inhibited by its products succinyl-CoA and NADH; a positive total energy balance in the cell has also an inhibitory effect on the enzymatic activity of KGDH [43]. ADP and Ca^2+^ play a role as allosteric activators of KGDH [44]. Also for KGDH, 2F-ANOVA revealed a significantly decreased activity (*p* < 0.05) in cardiac mitochondria of animals fed with LMgD; however, in this case, no significant effect (*p* = 0.36) of the *Slc41a1*^−/−^ genotype was detected (Figure 3A,B). A trend for interaction (*p* = 0.077) between Mg diet and *Slc41a1* genotype existed with regard to KGDH activity. The eROC curve analyses revealed 74.4% and 62.2% ability to discriminate KGDH activities according to type of Mg diet and *Slc41a1* genotype, respectively (Figure 3C,D); the considered uncertainties of the ROC curve estimates are given in Figure 3C,D.

### 2.2. Effect of Dietary Mg Content and A1 Genotype on Activities of ETC Complexes (Including F_1_F_o_-ATPase) in Lysates of Murine Mitochondria of Cardiac Cells

ETC is a redox cascade, downstream of Krebs cycle, consisting of protein complexes enabling the transport of electrons (to their final acceptor O_2_) coupled with the transport of H^+^ from matrix to inter-membrane space, resulting in build-up of a large inside-negative membrane potential on the inner mitochondrial membrane and in the production of ATP by CV/F_1_F_o_-ATPase. Only a paucity of information is available regarding any possible interference between mitochondrial Mg homeostasis, ETC, and consequent energy production and metabolism.

**Figure 4:**
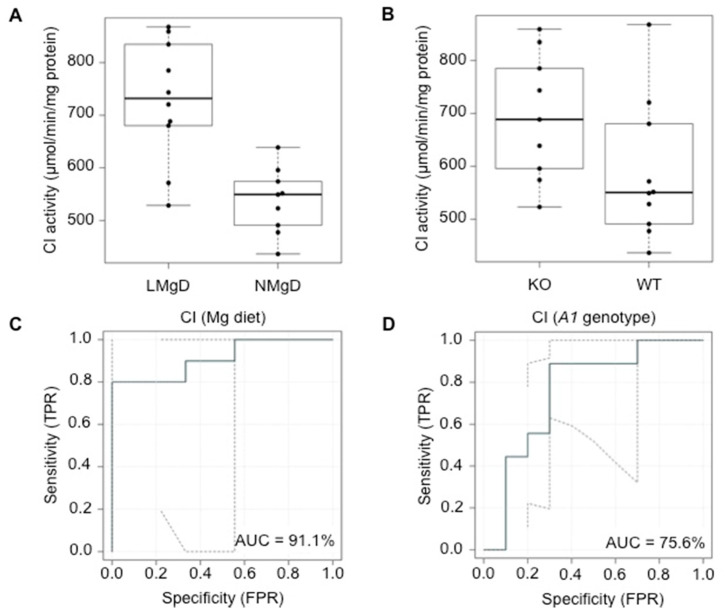
Impact of dietary Mg content and *Slc41a1* genotype on the activity of complex I (CI) in murine cardiac mitochondria. (**A**) Box-and-whisker plot overlaid with dot plot showing the influence of dietary Mg content (LMgD vs. NMgD) and (**B**) *Slc41a1* genotype (*Slc41a1*^−/−^ vs. *Slc41a1*^+/+^) on CI activities in murine mitochondria. For both, A and B, the original source data and corresponding medians are indicated for each group. (**C**,**D**) Empirical ROC (receiver operating characteristic) curves with the area under the ROC curve (AUC) for (**C**) the type of Mg diet as predictor of CI activity and (**D**) the *Slc41a1* genotype as predictor of CI activity. Abbreviations: *A1*, *Slc41a1*; FPR, false-positive rate; KO, knock-out; TPR, true-positive rate; WT, wild-type.

The 2F-ANOVA revealed a significant increasing effect (*p* < 0.001) of dietary Mg starvation on CI activity in cardiac mitochondria when the animals on LMgD and the animals on NMgD were compared balanced for *Slc41a1* genotype (Figure 4A). Irrespective of dietary regime, a significantly higher (*p* < 0.05) activity of CI was measured in cardiac mitochondria of *Slc41a*^−/−^ animals when compared with the CI activity in mitochondria of *Slc41a1*^+/+^ animals (Figure 4B). No significant interaction (*p* = 0.74) between dietary Mg content and *Slc41a1* genotype was detected for CI activity. Based on eROC curve analyses, the type of Mg diet and *Slc41a1* genotype had 91.1% and 75.6% ability to serve as discriminants/predictors of CI activities, respectively (Figure 4C,D); the uncertainties of the ROC curve estimates were considered and are given in Figure 4C,D.

**Figure 5:**
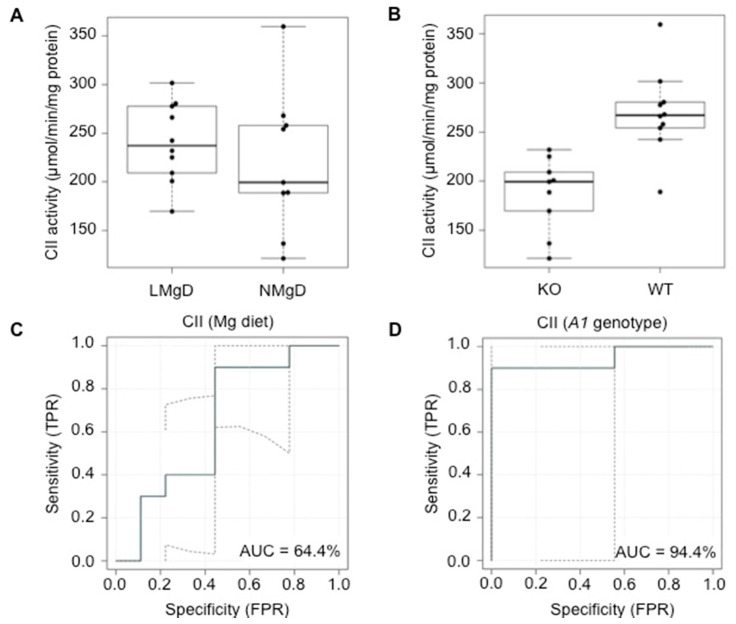
Impact of dietary Mg content and *Slc41a1* genotype on the activity of complex II (CII) in murine cardiac mitochondria. (**A**) Box-and-whisker plot overlaid with dot plot showing the influence of dietary Mg content (LMgD vs. NMgD) and (**B**) *Slc41a1* genotype (*Slc41a1*^−/−^ vs. *Slc41a1*^+/+^) on CII activity in murine mitochondria. For both, A and B, the original source data and corresponding medians are indicated for each group. (**C**,**D**) Empirical ROC (receiver operating characteristic) curves with the area under the ROC curve (AUC) for (**C**) the type of Mg diet as predictor of CII activity and (**D**) the *Slc41a1* genotype as predictor of CII activity. Abbreviations: *A1*, *Slc41a1*; FPR, false-positive rate; KO, knock-out; TPR, true-positive rate; WT, wild-type.

The 2F-ANOVA data analysis revealed no significant effect (*p* = 0.18) of the diet on CII activity in cardiac mitochondria when animals on LMgD and NMgD were compared (Figure 5A). A significant decreasing effect (*p* < 0.001) of the *Slc41a1*^−/−^ genotype on the CII activity was seen in cardiac mitochondria when compared with CII activity in mitochondria of *Slc41a1*^+/+^ animals (Figure 5B). No significant interaction (*p* = 0.32) between dietary Mg content and *Slc41a1* genotype was detected for CII activity. The eROC curve analyses revealed 64.4% and 94.4% ability of type of Mg diet and *Slc41a1* genotype to serve as discriminants/predictors of CII activities, respectively (Figure 5C,D); the uncertainties of the ROC curve estimates are given in Figure 5C,D.

**Figure 6:**
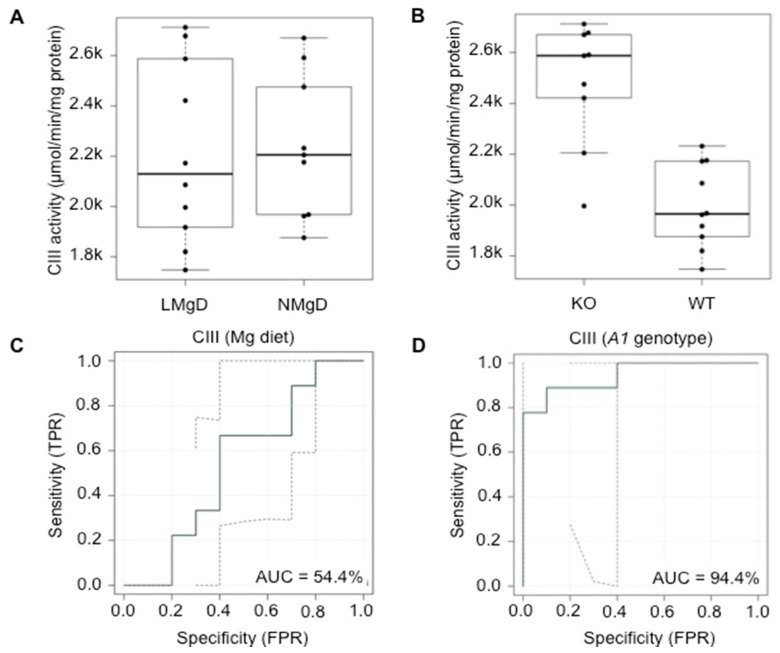
Impact of dietary Mg content and *Slc41a1* genotype on the activity of complex III (CIII) in murine cardiac mitochondria (**A**) Box-and-whisker plot overlaid with dot plot showing the influence of dietary Mg content (LMgD vs. NMgD) and (**B**) *Slc41a1* genotype (*Slc41a1*^−/−^ vs. *Slc41a1*^+/+^) on CIII activity in murine mitochondria. For both, A and B, the original source data and corresponding medians are indicated for each group. (**C**,**D**) Empirical ROC (receiver operating characteristic) curves with the area under the ROC curve (AUC) for (**C**) the type of Mg diet as predictor of CIII activity and (**D**) the *Slc41a1* genotype as predictor of CIII activity. Abbreviations: *A1*, *Slc41a1*; FPR, false-positive rate; KO, knock-out; TPR, true-positive rate; WT, wild-type.

The statistical analysis with 2F-ANOVA revealed no significant effect (*p* = 0.60) of the diet on CIII activity in heart mitochondria when the animals on LMgD and the animals on NMgD were compared balanced for genotype (Figure 6A). Irrespective of the type of Mg diet, a significant increasing effect (*p* < 0.001) of the *Slc41a1*^−/−^ genotype on the activity of CIII was measured in cardiac mitochondria when compared with the CIII activity in mitochondria of *Slc41a1*^+/+^ animals (Figure 6B). No significant (*p* = 0.66) interaction between Mg diet and *Slc41a1* genotype was determined for CIII activity. The 54.4% and 94.4% ability of the type of Mg diet and of *Slc41a1* genotype to discriminate/predict CIII activities were determined by eROC curve analyses, respectively (Figure 6C,D); the uncertainties of the ROC curve estimates are given in Figure 6C,D.

**Figure 7:**
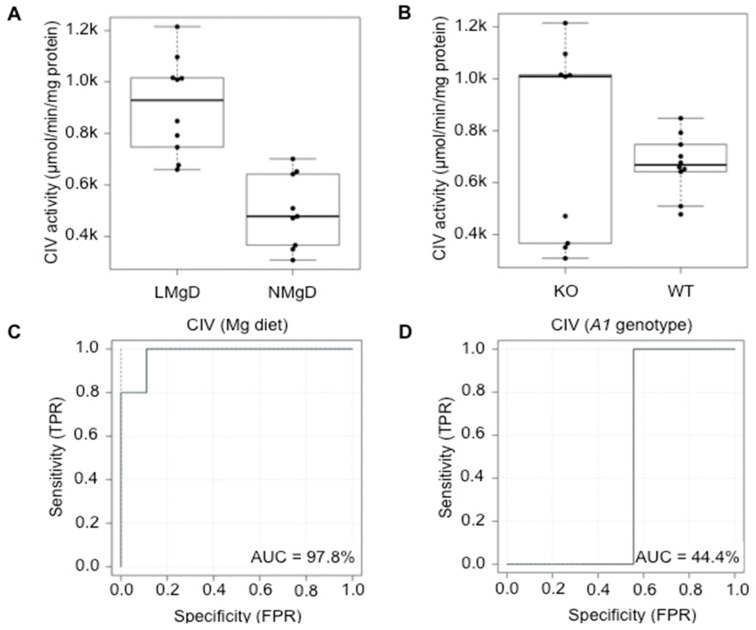
Impact of dietary Mg content and *Slc41a1* genotype on the activity of complex IV (CIV) in murine cardiac mitochondria. (**A**) Box-and-whisker plot overlaid with dot plot showing the influence of dietary Mg content (LMgD vs. NMgD) and (**B**) *Slc41a1* genotype (*Slc41a1*^−/−^ vs. *Slc41a1*^+/+^) on CIV activity in murine mitochondria. For both, A and B, the original source data and corresponding medians are indicated for each group. The interaction of data is presented in Figure A1. (**C**,**D**) Empirical ROC (receiver operating characteristic) curves with the area under the ROC curve (AUC) for (**C**) the type of Mg diet as predictor of CIV activity and (**D**) the *Slc41a1* genotype as predictor of CIV activity. Abbreviations: *A1*, *Slc41a1*; FPR, false-positive rate; KO, knock-out; TPR, true-positive rate; WT, wild-type.

**Figure A1:**
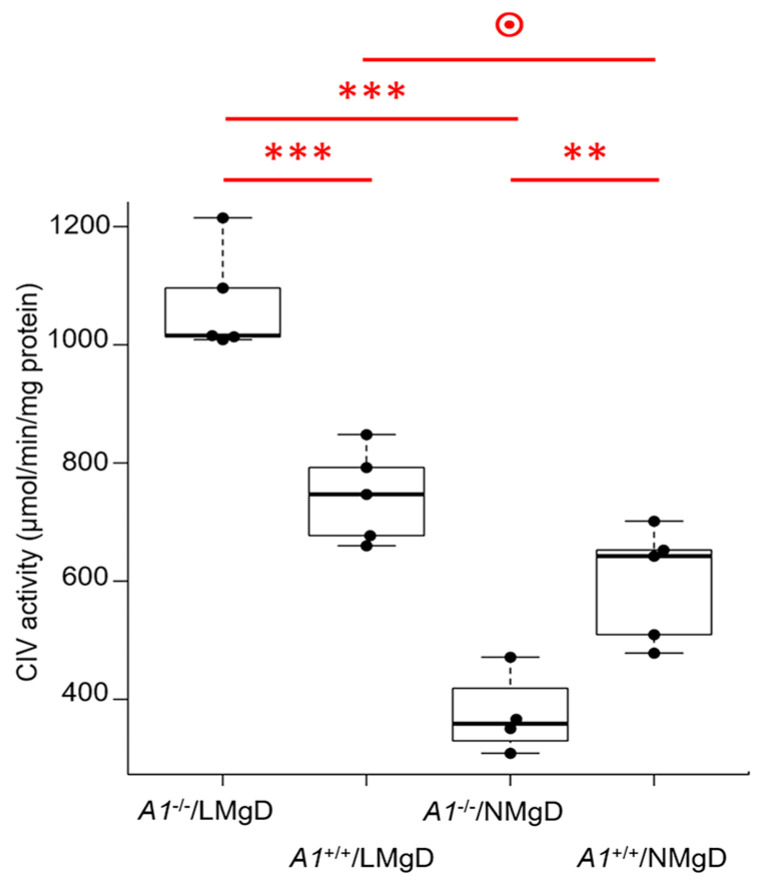
*Post-hoc* Tukey’s range test/CIV. The CIV activities presented as box-and-whisker plot overlaid with dot plot; dots represent raw input values for each analyzed group (*Slc41a1*^−/−^ animals on LMgD, *Slc41a1*^+/+^ animals on LMgD, *Slc41a1*^−/−^ animals on NMgD, and *Slc41a1*^+/+^ animals on NMgD); ** *p* < 0.01, *** *p* < 0.001. Abbreviations: ⦿, trend towards significance (*p* < 0.1); *A1*, *Slc41a1*; CIV, complex IV; LMgD, low Mg diet; NMgD, normal Mg diet.

For CIV (cytochrome c oxidase) activity, significant increasing effects of LMgD vs. NMgD (*p* < 0.001; Figure 7A) and *Slc41a1*^−/−^ vs. *Slc41a1*^+/+^ genotype (*p* < 0.05; Figure 7B) were detected in cardiac mitochondria. However, a highly significant interaction (*p* < 0.001) between dietary Mg content and *Slc41a1* genotype indicated a strong interdependence of the two factors. As such, no significant difference (*p* = 0.064) of CIV activity was detected in cardiac mitochondria when *Slc41a1*^+/+^ animals on LMgD were compared with *Slc41a1*^+/+^ animals on NMgD (LMgD 745 ± 20 nmol/min/mg protein vs. NMgD 597 ± 25 nmol/min/mg protein; Figure A1). By contrast, the CIV activity was increased in cardiac mitochondria by 186.1% (*p* < 0.001) in *Slc41a1*^−/−^ animals on LMgD when compared to *Slc41a1*^−/−^ animals on NMgD (LMgD 1070 ± 22 nmol/min/mg protein vs. NMgD 374 ± 20 nmol/min/mg protein; Figure A1). Moreover, cardiac mitochondria of *Slc41a1*^−/−^ animals had 37.4% lower CIV activity on NMgD (*p* < 0.01) and 43.6% higher CIV activity on LMgD (*p* < 0.001) when compared with cardiac mitochondria from *A1*^+/+^ animals (Figure A1).

The eROC analyses revealed 97.8% and 44.4% ability of the type of Mg diet and of *Slc41a1* genotype to serve as discriminants/predictors of CIV activities, respectively (Figure 7C,D); the uncertainties of the ROC curve estimates are given in Figure 7C,D.

**Figure 8:**
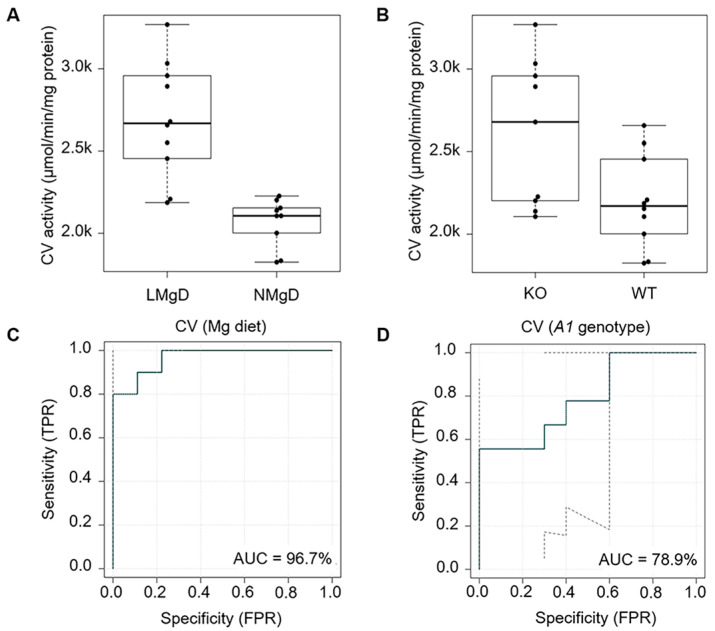
Impact of dietary Mg content and *Slc41a1* genotype on the activity of complex V/F_1_F_o_-ATPase (CV) in murine cardiac mitochondria. (**A**) Box-and-whisker plot overlaid with dot plot showing the influence of dietary Mg content (LMgD vs. NMgD) and (**B**) *Slc41a1* genotype (*Slc41a1*^−/−^ vs. *Slc41a1*^+/+^) on CV activity in murine mitochondria. For both, A and B, the original source data and corresponding medians are indicated for each group. The interaction of data is presented in Figure A2. (**C**,**D**) Empirical ROC (receiver operating characteristic) curves with the area under the ROC curve (AUC) for (**C**) the type of Mg diet as predictor of CV activity and (**D**) the *Slc41a1* genotype as predictor of CV activity. Abbreviations: *A1*, *Slc41a1*; FPR, false-positive rate; KO, knock-out; TPR, true-positive rate; WT, wild-type.

**Figure A2:**
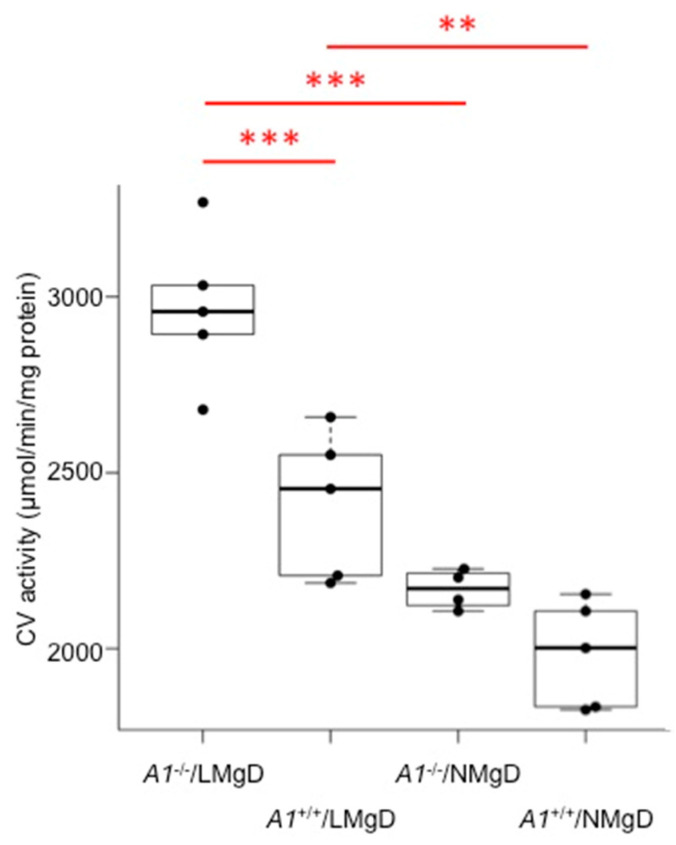
*Post-hoc* Tukey’s range test/CV. The CV activities presented as box-and-whisker plot overlaid with dot plot; dots represent raw input values for each analyzed group (*Slc41a1*^−/−^ animals on LMgD, *Slc41a1*^+/+^ animals on LMgD, *Slc41a1*^−/−^ animals on NMgD, and *Slc41a1*^+/+^ animals on NMgD); ** *p* < 0.01, *** *p* < 0.001. Abbreviations: *A1*, *Slc41a1*; CV, complex V; LMgD, low Mg diet; NMgD, normal Mg diet.

Similar to CIV, the 2F-ANOVA revealed significant increasing effects of the LMgD vs. NMgD (*p* < 0.001; Figure 8A) and *Slc41a1*^−/−^ vs. *Slc41a1^+/+^* genotype (*p* < 0.001; Figure 8B) on CV (F_1_F_o_-ATPase) activity in mitochondria. Additionally, significant interaction (*p* < 0.05) between dietary Mg content and *Slc41a1* genotype was found. The multiple-comparison *post-hoc* test for individual groups verified a significant increase in CV activity of cardiac mitochondria by 17.8% and 26.9% when comparing either KO animals (*p* < 0.001; LMgD 2967 ± 54 nmol/min/mg protein vs. NMgD 2168 ± 16 nmol/min/mg protein) or WT animals (*p* < 0.01; LMgD 2412 ± 53 nmol/min/mg protein vs. NMgD 1984 ± 38 nmol/min/mg protein) on LMgD with those on NMgD, respectively (Figure A2). For genotype, however, an significant increase of CV activity by 18.7% was only observed within LMgD when switching from *Slc41a1*^+/+^ to *Slc41a1*^−/−^ genotype (*p* < 0.001); whereas, the genotype effect was not significant (*p* = 0.42) within NMgD (Figure A2).

The eROC analyses revealed 96.7% and 78.9% ability of the type of Mg diet and of *Slc41a1* genotype to discriminate/predict activities of CV, respectively (Figure 8C,D); the uncertainties of the ROC curve estimates are given in Figure 8C,D.

### 2.3. Random Forest Machine-Learning (RFM-L) Analysis of the Activities of Selected Krebs Cycle Enzymes and ETC Complexes as Potential Predictors/Discriminators between LMgD and NMgD and between Slc41a1^−/−^ and Slc41a1^+/+^ Genotypes

**Figure 9:**
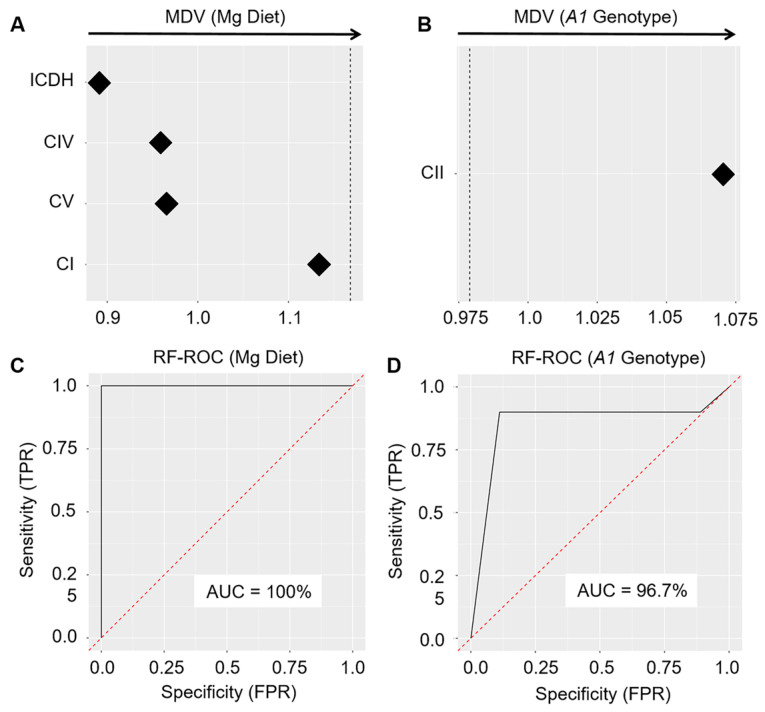
Importance plots depicting prioritization of selected predictors according to their discriminative ability between the types of Mg diet (LMgD vs. NMgD; (**A**) or the *Slc41a1* genotypes (*Slc41a1*^−/−^ vs. *Slc41a1*^+/+^; (**B**). In panela A and B, the dashed lines separates the well-suitable predictors (left from the line) from the less-suitable predictors (right from the line). ROC (receiver operating characteristic) curves with the area under the ROC curve (AUC) for *RFM-L* algorithm with (**C**) ICDH, CI, CIV, and CV as joint predictors of the type of Mg diet, and with (**D**) CII as predictor of the *Slc41a1* genotype. Abbreviations: *A1*, *Slc41a1*; FPR, false positive rate; MDV, minimal depth of a variable; RFM-L, random forest machine-learning; TPR, true positive rate.

In order to analyze the activities of ACON, ICDH, KGDH, CI, CII, CIII, CIV, and CV/F_1_F_o_-ATPase as potential predictors/discriminators between the types of Mg diet fed to MK-1 mice (LMgD vs. NMgD) and between the *Slc41a1* genotypes of the animals (*Slc41a1*^−/−^ vs. *Slc41a1*^+/+^), we trained the RFM-L algorithm using our data. The algorithm evaluated the discriminative importance of individual activities of the tested Krebs cycle enzymes and ETC complexes by a technical construct known as graph depth [45]. The predictive ability of these enzymes/enzyme complexes was visualized by ROC curves and quantified by AUC. A perfect discriminative ability of predictors is associated with 100% AUC; 50% AUC (or less) corresponds to no discriminative ability [45]. The best discriminative ability between mitochondria isolated from the hearts of animals fed with LMgD and mitochondria isolated from the hearts of animals fed with NMgD (irrespective of their *Slc41a1* genotype) were computed for the activity of ICDH followed by the activities of CIV > CV > CI (Figure 9A). The importance plot revealed the activity of CII (Figure 9B) as the only suitable individual discriminator between mitochondria isolated from hearts of *Slc41a1*^−/−^ mice and the mitochondria from hearts of *Slc41a1*^+/+^ mice (irrespective of the content of Mg in their diet). The RFM-L algorithm trained in the mode in which the activities of ICDH, CI, CIV, and CV served as joined predictors led to an ROC curve with 100% AUC; thus, the joint activities of ICDH, CI, CIV, and CV can serve as a perfect predictor/discriminator between LMgD and NMgD (Figure 9C). Training of the RFM-L algorithm in the mode in which the activity of CII served as a predictor led to an ROC curve with 96.7% AUC; thus, the activity of CII could be considered as a near perfect predictor/discriminator between the *Slc41a1*^−/−^ and *Slc41a1*^+/+^ genotypes (Figure 9D).

### 2.4. A Brief Summary of the Results

**Figure A3:**
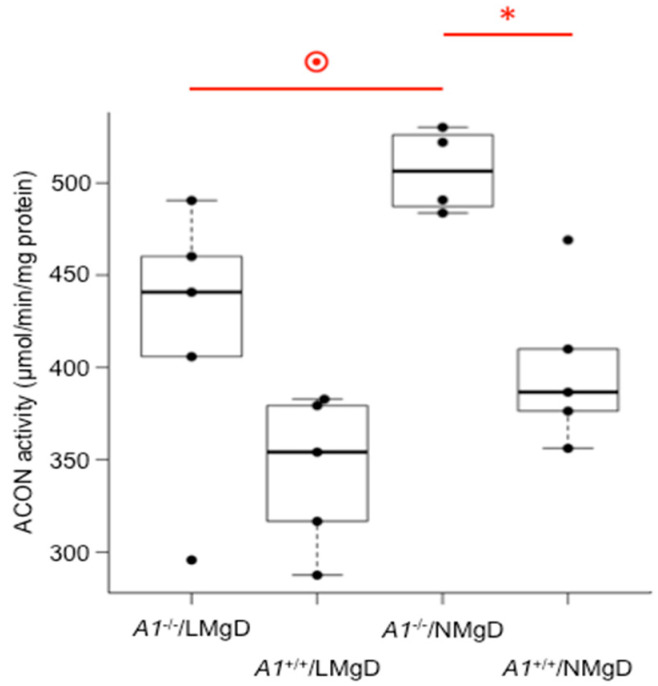
*Post-hoc* Tukey’s range test/ACON. The ACON activities presented as box-and-whisker plot overlaid with dot plot; dots represent raw input values for each analyzed group (*Slc41a1*^−/−^ animals on LMgD, *Slc41a1*^+/+^ animals on LMgD, *Slc41a1*^−/−^ animals on NMgD, and *Slc41a1*^+/+^ animals on NMgD); * *p* < 0.05. Abbreviations: ⦿, trend towards significance (*p* < 0.1); *A1*, ACON, aconitate hydratase; *Slc41a1*; LMgD, low Mg diet; NMgD, normal Mg diet.

**Figure A4:**
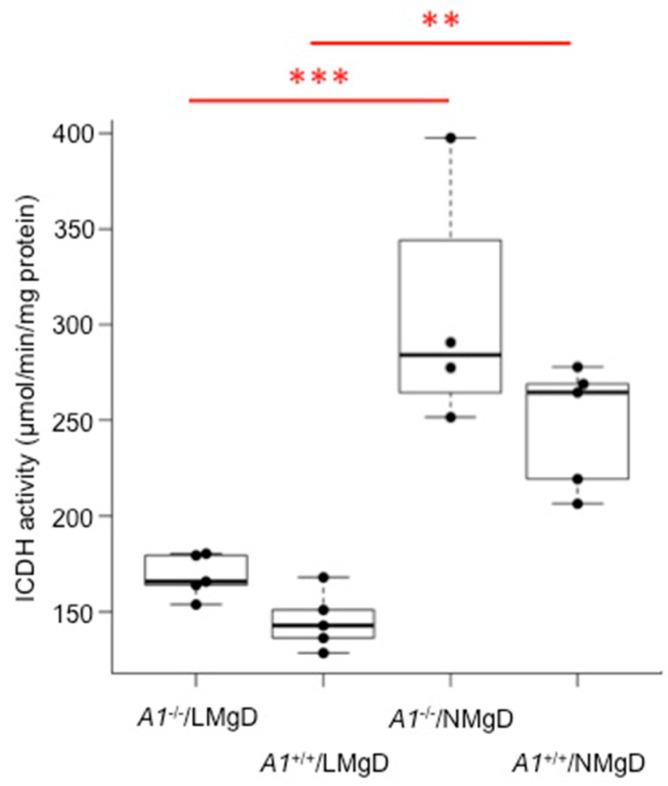
*Post-hoc* Tukey’s range test/ICDH. The ICDH activities presented as box-and-whisker plot overlaid with dot plot; dots represent raw input values for each analyzed group (*Slc41a1*^−/−^ animals on LMgD, *Slc41a1*^+/+^ animals on LMgD, *Slc41a1*^−/−^ animals on NMgD, and *Slc41a1*^+/+^ animals on NMgD); ** *p* < 0.01, *** *p* < 0.001. Abbreviations: *A1*, *Slc41a1*; ICDH, isocitrate dehydrogenase; LMgD, low Mg diet; NMgD, normal Mg diet.

**Figure A5:**
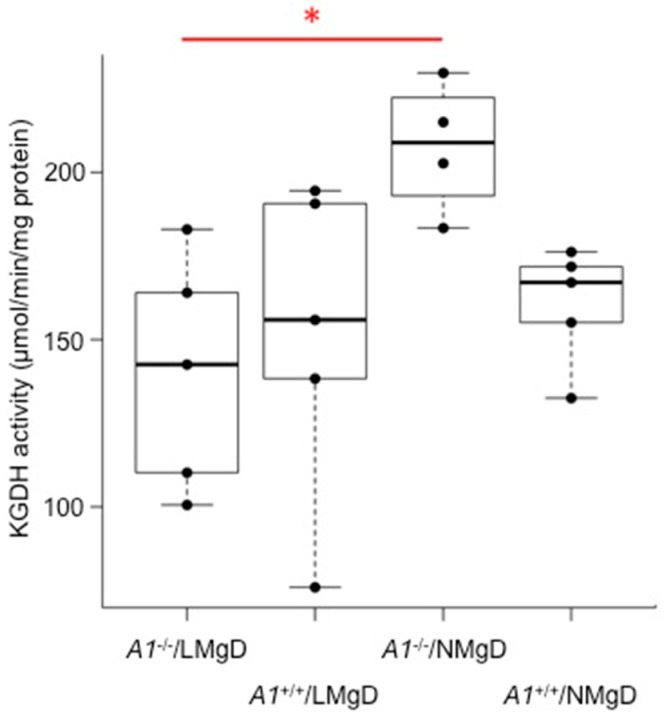
*Post-hoc* Tukey’s range test/KGDH. The KGDH activities presented as box-and-whisker plot overlaid with dot plot; dots represent raw input values for each analyzed group (*Slc41a1*^−/−^ animals on LMgD, *Slc41a1*^+/+^ animals on LMgD, *Slc41a1*^−/−^ animals on NMgD, and *Slc41a1*^+/+^ animals on NMgD); * *p* < 0.05. Abbreviations: *A1*, *Slc41a1*; KGDH, α-ketoglutarate dehydrogenase, LMgD, low Mg diet; NMgD, normal Mg diet.

**Figure A6:**
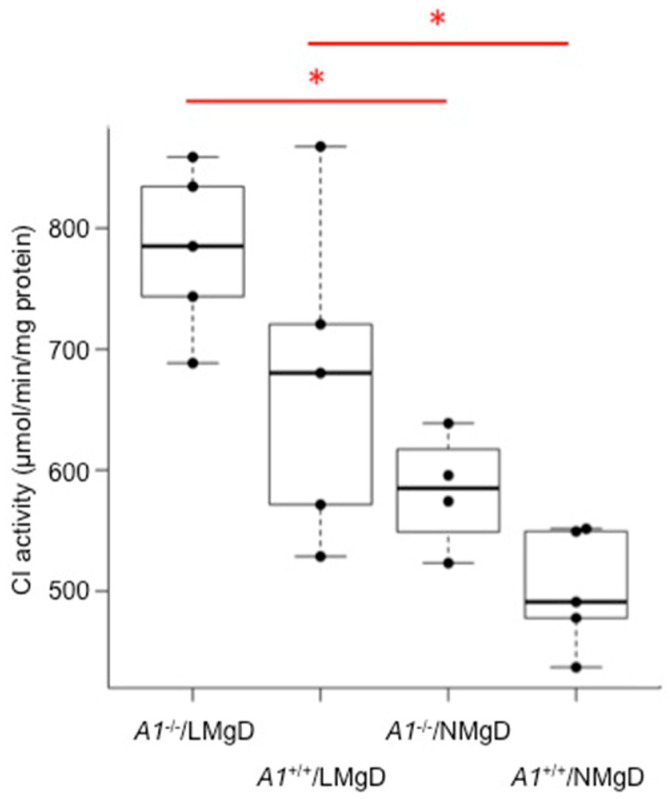
*Post-hoc* Tukey’s range test/CI. The CI activities presented as box-and-whisker plot overlaid with dot plot; dots represent raw input values for each analyzed group (*Slc41a1*^−/−^ animals on LMgD, *Slc41a1*^+/+^ animals on LMgD, *Slc41a1*^−/−^ animals on NMgD, and *Slc41a1*^+/+^ animals on NMgD); * *p* < 0.05. Abbreviations: *A1*, *Slc41a1*; CI, complex I; LMgD, low Mg diet; NMgD, normal Mg diet.

**Figure A7:**
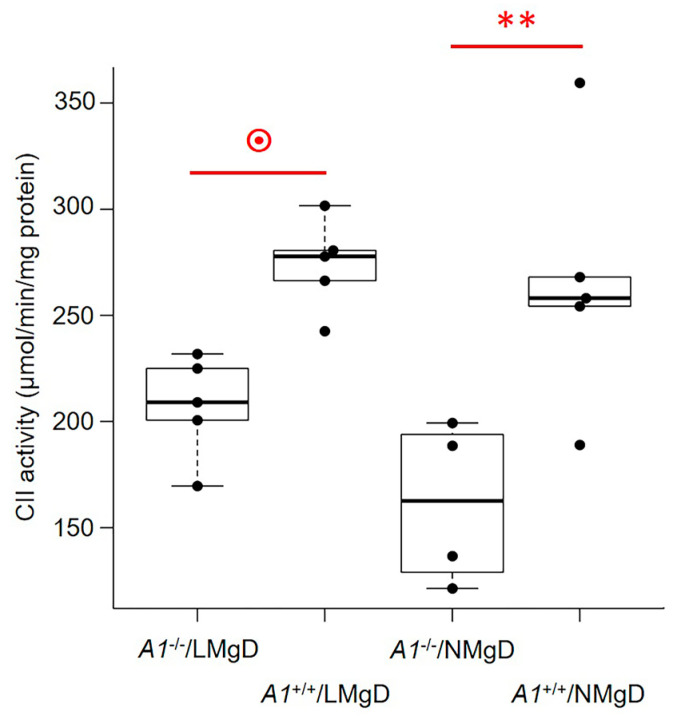
*Post-hoc* Tukey’s range test/CII. The CII activities presented as box-and-whisker plot overlaid with dot plot; dots represent raw input values for each analyzed group (*Slc41a1*^−/−^ animals on LMgD, *Slc41a1*^+/+^ animals on LMgD, *Slc41a1*^−/−^ animals on NMgD, and *Slc41a1*^+/+^ animals on NMgD); ** *p* < 0.01. Abbreviations: ⦿, trend towards significance (*p* < 0.1); *A1*, *Slc41a1*; CII, complex II; LMgD, low Mg diet; NMgD, normal Mg diet.

**Figure A8:**
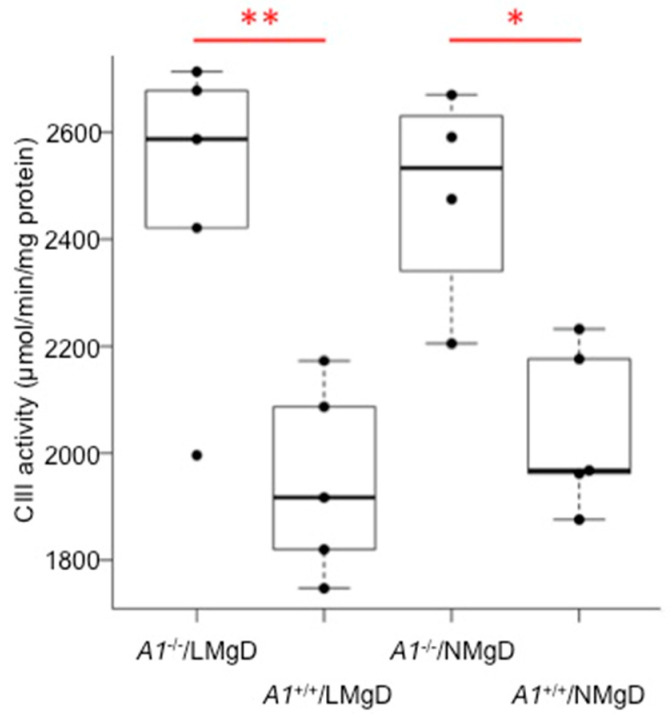
*Post-hoc* Tukey’s range test/CIII. The CIII activities presented as box-and-whisker plot overlaid with dot plot; dots represent raw input values for each analyzed group (*Slc41a1*^−/−^ animals on LMgD, *Slc41a1*^+/+^ animals on LMgD, *Slc41a1*^−/−^ animals on NMgD, and *Slc41a1*^+/+^ animals on NMgD); * *p* < 0.05, ** *p* < 0.01. Abbreviations: *A1*, *Slc41a1*; CIII, complex III; LMgD, low Mg diet; NMgD, normal Mg diet.

The results are briefly summarized in Table 1. The upper part of the table compiles outcome of 2F-ANOVA, while the bottom part (on gray background) summarizes outcomes of *post-hoc* Tukey multiple comparisons of group means. For CIV and CV the significant interaction between *Slc41a1* genotype and Mg diet has been identified and for both also the data resulting from *post-hoc* Tukey test were previously provided. To obtain the complex information carried in *post-hoc* analyze, they were performed also for each enzyme/complex, for which no significant interaction between *Slc41a1* genotype and Mg diet has been identified (ACON, ICDH, KGDH, CI, CII and CIII). The outcomes of these analyzes are provided in Appendix A (Figure A3, Figure A4, Figure A5, Figure A6, Figure A7 and Figure A8).

## 3. Discussion

Disturbed intracellular Mg homeostasis and chronic systemic Mg deficiency have been linked with cardiovascular complications [29]. CVD have further been associated with disturbed functions of mitochondria in cardiac cells [46]. Only recently, Mg has been revealed to play an essential role in the maintenance of mitochondrial homeostasis [20,32,47]. However, the molecular background behind the network between Mg homeostasis, mitochondrial function, and CVD is not as yet fully understood.

Here, we demonstrate that the Na^+^/Mg^2+^ exchanger SLC41A1 and dietary Mg intake are essential regulators of mitochondrial function in mice. Low Mg^2+^ diets were associated with reduced Krebs cycle activity and a compensatory increase in activity of ETC components in mitochondrial lysates form mouse hearts. Moreover, the effect of SLC41A1 on mitochondrial function was shown, indicating a close relationship between plasma membrane Mg^2+^ transport and energy metabolism.

A plethora of molecular components of the energy-producing machinery in mitochondria are dependent on Mg^2+^ [31,32,33,34,35,36,37,38,48,49]. Our data show that the type of Mg diet, i.e., Mg *per se*, influences the activities of ACON, ICDH, KGDH, CI, CIV, and CV in cardiomyocytes (Table 1). Indeed, Mg^2+^ is required for the enzymatic activity of ICDH and of KGDH [34,42]. Panov and Scarpa [34] showed that either Ca^2+^ or Mg^2+^ increase the activity of KGDH, and that the effects are additive if the [Ca^2+^] and [Mg^2+^] are below 0.1 mM and 1 mM, respectively. Moreover, Garfinkel and Garfinkel [31], as early as 1985, demonstrated that Mg is an important coherent controller of glycolysis and the Krebs cycle. Therefore, we assumed that a longer-lasting (2-week) Mg-starvation would negatively impact ICDH and KGDH activity in the mitochondria of cardiac cells, i.e., that it would negatively influence the direct energetic outcome and the production of ETC substrates in the Krebs cycle.

In our experimental setting, the depletion of dietary Mg led to decreases of the activities of all Krebs cycle enzymes measured (i.e., ACON, ICDH and KGDH) in cardiac mitochondria of both *Slc41a1*^−/−^ *and Slc41a1^+/+^* animals (Table 1). The lower activities of ACON, ICDH and KGDH can be explained by lower [Mg] in the mitochondrial matrix as it conforms with the reduction of most Krebs cycle metabolites in mitochondria observed after abolishing the influx of Mg into mitochondria via Mrs2 [20,39]. The new finding of the present study was that this can be reversed by knock-out of *Slc41a1*. When balanced for the Mg content of diet, the lack of functional Na^+^/Mg^2+^ exchanger SLC41A1 in cardiac cells had obvious positive effects on ACON, ICDH and KGDH. In the case of KGDH, this effect was only evident with NMgD, though there was a trend for interaction between the type of Mg diet and the *Slc41a1* genotype. For ACON and ICDH, however, no interaction was identified between type of Mg diet and the *Slc41a1* genotype, indicating that these factors superimpose on each other independently. Therefore, our data show clearly that the chronic lack of dietary Mg has obvious negative effects on ACON, and ICDH and KGDH, the key rate-limiting and regulatory components of Krebs cycle [42,43]. For the understanding of the present results, it is important to note that they represent the outcome of chronic dietary depletion of Mg on enzyme activity and not an effect of acute depletion of Mg^2+^ as cofactor during the actual measurement of enzyme activity. Enzyme activity measurements were done with the sufficient Mg concentration (as indicated in Material and Methods). Thus a decrease in enzyme activity measured with our assay could be aggravated further by an actual decrease of intracellular Mg^2+^ concentration in vivo. Vice versa, our results further show that knock-out of *Slc41a1* can at least partially rescue the negative effects of Mg starvation on Krebs cycle enzymes in cardiac mitochondria.

Yamanaka and colleagues [20] have proposed that the lack of intra-mitochondrial Mg^2+^ suppresses the efflux of ATP from the mitochondria (most probably conducted via the ATP-Mg/P_i_ carrier [21]) resulting in ATP accumulation in the mitochondrial matrix. Excess ATP in mitochondria is well known to inhibit ICDH and KGDH in Krebs cycle; thus, a lack of Mg^2+^ might indirectly contribute to the suspension of ATP production by mitochondria [20,50]. This mechanism is poorly researched but would give Mg^2+^ the role of a central coordinator of the key mitochondrial processes, namely Krebs cycle, ETC, and ADP/ATP translocation [20]. Our data seem to fully support Yamanaka’s hypothesis [20].

In our experiments, the chronic lack of dietary Mg evidently led to increased activity of CI, CIV, and CV in lysates of cardiac mitochondria (Table 1). The latter applied to both genotypes for CI and CV; whereas, it applied to only *Slc41a1*^−/−^ animals in the case of CIV. These data are generally compatible with data from male broiler chickens fed with LMgD (Mg = 1.2 g/kg dry matter) or control diet (Mg = 2.4 g/kg dry matter) for 6 weeks [51]. Compared with the control, the muscle [Mg] of broiler chickens from the LMgD group decreased by 9.5%. In parallel, activities of CII and CIII of ETC increased by 23% and 35% in the broilers on LMgD, respectively. Based on these findings, the authors concluded that the feeding of broilers with LMgD induced higher activities of mitochondrial ETC [51]. It remains to be answered by further studies why the ETC complexes CI, CIV, and CV were more active in mice fed with LMgD in our study, whereas CII and CIII were more active in the previous study of Liu and coworkers [51]. Possible factors include origin/species (mouse vs. chicken), target organ (heart vs. skeletal muscle), and duration of starvation (2 weeks vs. 6 weeks). Another important factor might be the content of Mg in the LMgD, which was, in our case, six times lower than that in the study of Liu and colleagues [51].

Teleologically, the increased activity of the ETC could represent an attempt to compensate for the reduced activity of the Krebs cycle in order to maintain energy provision during Mg-deficiency. It has to be considered that our data (and also of Liu and coworkers [51]) have been acquired in vitro, when substrate availability is not limiting the reactions. If we see increasing activities of certain ETC complexes in our in vitro experimental settings after Mg starvation, but in the presence of sufficient Mg and substrates, this may not similarly apply in vivo where Mg and substrates are limiting. As such, we hypothesize that cells overexpress ETC complexes, to compensate for decreased performance caused by the lack of substrates and Mg^2+^. For example, Mg^2+^-depleted cytochrome c oxidase retains only 40% activity of the native, Mg^2+^-containing enzyme [48] and would thus require overexpression during Mg^2+^ deficiency to secure near to normal performance of ETC. Upon our experimental conditions, when above physiological amounts of Mg^2+^ [39,52] and excessive amounts of substrates are provided, the overexpression of particular ETC complexes then reflects as gain of their activities above those in controls.

The role of SLC41A1 for ETC is less clear because in most instances the knock-out of *Slc41a1* did not counteract the stimulation of ETC by LMgD but rather co-stimulated the activity of certain ETC complexes. The activities of CIII, CIV, and CV were increased in the cardiac mitochondria of *Slc41a1*^−/−^ animals fed with LMgD (Table 1). The heart mitochondria of *Slc41a1*^−/−^ animals fed with NMgD exhibit significantly increased activities of CIII and decreased activities of CII and CIV when compared with *Slc41a1*^+/+^ animals (Table 1). Irrespective of the type of Mg diet, we have observed increased activities of CI, CIII, CIV, and CV and the decreased activity of CII in *Slc41a1*^−/−^ compared with *Slc41a1*^+/+^ animals (Table 1). These data make it clear that the presence or absence, and thus the functionality, of SLC41A1 influences mitochondrial processes involved in energy production.

Finally, we utilized RFM-L with the aim of identifying, among the tested enzymes and ETC complexes, those that might reliably distinguish between the type of Mg diet or the *Slc41a1* genetic background. Following the priority pre-analysis, the activities of ICDH, CI, CIV, and CV jointly qualified as perfect markers able to distinguish between samples from LMgD-fed and NMgD-fed animals with 100% reliability. Importantly, the outcomes of the RFM-L analysis and of the standard frequentist statistics approach overlap. RFM-L analysis further identified the activity of CII as a 96.7% reliable marker able to distinguish between *Slc41a1*^−/−^ and *Slc41a1*^+/+^ animals. In this case, the outcome is once again in agreement with the results of the 2F-ANOVA.

The present study provided several lines of molecular and biochemical proof that dietary Mg content and SLC41A1 functionality are crucial for the energy-production in cardiac mitochondria. To extrapolate more clearly on their physiological and disease relevance, future studies on the effects of dietary Mg and SLC41A1 function on cellular energy metabolism in cardiac cells (or any other tissues) should extend investigations to monitoring of additional parameters such as oxygen consumption/mitochondrial respiration, ATP production, ROS production and expression of markers of the mitochondrial oxidative stress, as well as measurement of the mitochondrial membrane potential (ΔΨ_m_). Structural changes of mitochondrial architecture related to changed activities of the energy metabolism constituents are also possible and should be examined [53].

## 4. Materials and Methods

### 4.1. Development of Slc41a1 Knock-Out Mouse

**Figure 10:**
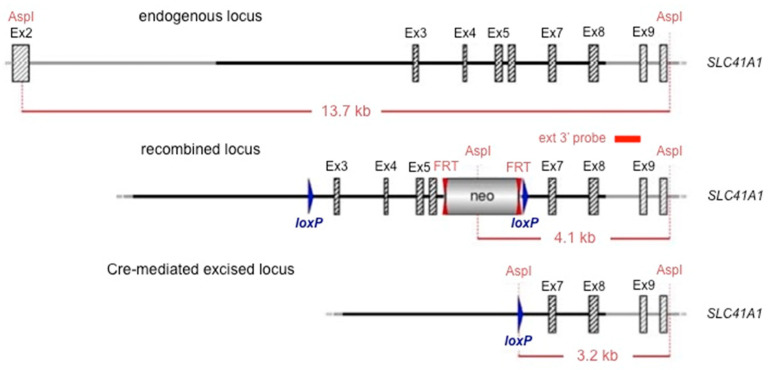
Scheme of Cre- or Flp-excision at the recombined *Slc41a1* locus. Abbreviations: AspI, restriction endonuclease cutting palindrome 5′GACNNNGTC3′; Ex, exon; FLP, flippase; FRT, flippase recognition target; *loxP*, locus of X-over P1. The position of the external 3′ probe used for Southern blot detection is indicated.

Constitutive KO *Slc41a1*^−/−^ mice (strain MK-1), derived from the C57BL/6 mouse strain, were developed in cooperation with GenOway (Lyon, France). The exon/intron organization of the gene was established based on the *Slc41a1* cDNA sequence AK154819. The mouse *Slc41a1* is located on chromosome 1 and extends over 20.8 kb. The gene comprises 11 exons. The ATG translation initiation and the STOP codons are located in exons 2 and 11, respectively. A 2126-bp 3′-UTR has been recognized. The murine *A1* encodes for a 512 amino acid open reading frame. To generate *Slc41a1*^−/−^ mice (based on the bioinformatics analysis of *Slc41a1*), we decided to delete *Slc41a1* exons 3 to 6 (Figure 10). The recombinant *Slc41a1* comprising *loxP* (locus of X-over P1 sites) and *FRT* (flippase (Flp) recognition target)-neo-*FRT* cassette was constructed, allowing for the utilization of Cre-*lox* and Flp-*FRT* technologies, respectively (Figure 10). The strategy resulted in the deletion of a 2800-bp fragment comprising coding sequences encoding for one of the two MgtE domains of the protein. Splicing of exons 2 to 7 led to a frame shift, which resulted in a premature stop codon in exon 8. Via the stage of recombinant ES cells, blastocyst injection, and generation of chimeras, we developed a floxed mouse line suitable for the generation of a conditional KO model for *Slc41a1*. This mouse line was further mated with deleter mice, which constitutively express the Cre recombinase, to generate a constitutive *Slc41a1*^−/−^ KO model in which the genetic manipulation is present in all cells of the animal.

### 4.2. Animal Breeding, Housing, and Euthanization

The study was approved by the Animal Ethics Board of the Radboud University Nijmegen (RU DEC 2015-0112; 22-02-2016) and by the Dutch Central Commission for Animal Experiments (AVD103002016382; 22-02-2016). In total, ten *Slc41a1*^+/+^ and ten *Slc41a1*^−/−^ male mice aged between 8–12 weeks were selected for this study. The animals were individually housed in metabolic cages for 48 h (24 h adaptation, 24 h sampling) prior to the collection of urine and feces. Subsequently, the mice were randomly divided into two groups of five animals and fed with a normal (0.23% (*w*/*w*)) or with a low (0.02% (*w*/*w*)) Mg^2+^ synthetic diet (Ssniff Spezialdiäten, Soest, Germany; *n* = 5 per group per genotype) for two weeks. Researcher and animal caretakers were blinded for the Mg^2+^ content of the experimental diets throughout the experiment. Animals were sacrificed via exsanguination under isoflurane anesthesia. The heart and other organs were collected for further analyses. One *Slc41a1*^−/−^ mouse from NMgD group died prematurely before completion of the experiments.

### 4.3. Isolation of Cardiac Mitochondria

Frozen powdered tissue of the whole murine heart (about 150 mg) was thawed in 10 volumes of ice-cold homogenization buffer (30 mM KH_2_PO_4_, 5 mM EDTA, 0.3 M sucrose; pH 7.0), with 0.3 mM phenylmethylsulfonyl fluoride as a protease inhibitor, and homogenized 5× at 1200 rpm in 25 s/20 s intervals with a polytetrafluoroethylene pestle in a Potter-Elvehjem homogenizer. The mitochondrial fraction was isolated from individual tissue homogenates (1 mL aliquots) by differential centrifugation at 3200× *g* for 10 min at 4 °C, and the supernatant obtained was further centrifuged at 10,000× *g* for 40 min at 4 °C. The resulting mitochondrial fraction was resuspended in 0.2 mL standard solution (30 mM imidazole, 60 mM KCl, and 2 mM MgCl_2_) and stored at −70 °C for further experiments.

**Figure A9:**
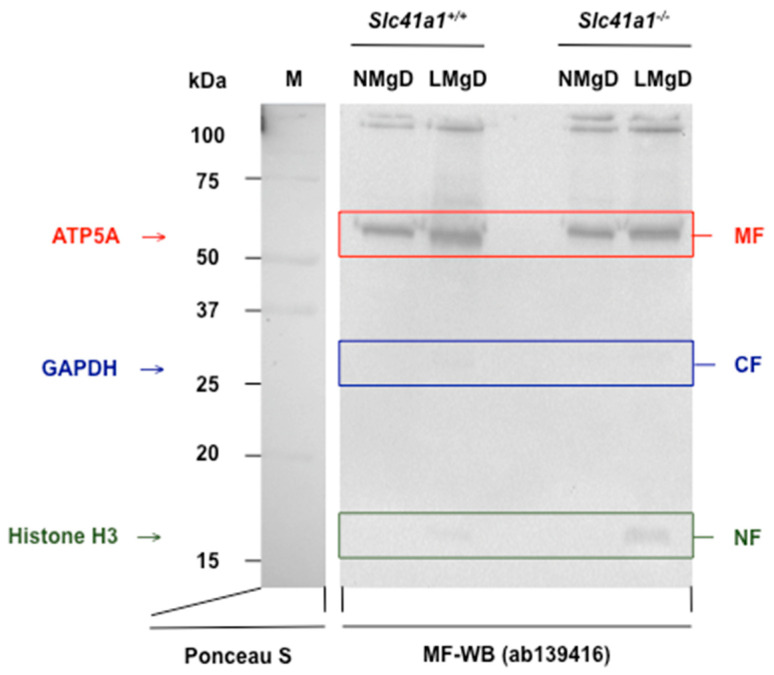
Representative Western blot (WB) documenting purity of isolated mitochondria from cardiac cells of *Slc41a1^+/+^* and *Slc41a1*^−/−^ animals on either NMgD or LMgD. Mitochondria fraction Western blot (MF-WB) cocktail (Abcam ab139416; containing antibodies against ATP5A (MW = 60 kDa; mitochondrial fraction (MF) marker, red color code), GAPDH (MW = 36 kDa; cytosolic fraction (CF) marker; blue color marker), and histone H3 (MW = 17 kDa; nuclear fraction (NF) marker; green color code). Abbreviations: LMgD, low Mg diet; M, marker; NMgD, normal Mg diet.

The purity of the mitochondrial fraction was estimated by immunodetection (anti-mitochondria fraction Western blot cocktail antibody, ab139416, Abcam, Cambridge, UK) involving the positive detection of ATP5A (mitochondrial marker) and the minimal/no presence of cytosolic GAPDH or nuclear marker histone H3 (Figure A9).

Protein concentrations were determined with a DC Protein assay (Bio-Rad Laboratories, Hercules, CA, USA). Bovine serum albumin was used as a standard.

### 4.4. Determination of Krebs Cycle Enzymes Activities

Activities of Krebs cycle enzymes were evaluated after exposure of cardiac mitochondria (diluted in 25.0 mM KH_2_PO_4_ and 0.5 mM EDTA, pH 7.25; containing 0.01% Triton X-100) to sonication for 30 s in a water bath. All assays were performed at room temperature, and enzyme activities were determined at 340 nm (*ε* = 6.2 mM^−1^·cm^−1^) according to the modified methods of Nulton-Persson and Szweda [54]. ACON activity was assayed as the rate of NADP^+^ reduction by ICDH upon the addition of 5.0 mM sodium citrate, 0.6 mM MgCl_2_, 0.2 mM NADP^+^, and 1.0 unit/mL ICDH to sonicated mitochondria (0.03 mg/mL protein). ICDH activity was assayed as the rate of NAD^+^ reduction upon the addition of 5.0 mM MgCl_2_, 0.04 mM rotenone, 2.5 mM isocitrate, and 1.0 mM NAD^+^ to 0.05 mg/mL mitochondrial protein. KGDH activity was assayed as the rate of NAD^+^ reduction upon the addition of 5.0 mM MgCl_2_, 0.04 mM rotenone, 2.5 mM α-ketoglutarate, 0.1 mM coenzyme A, 0.2 mM thiamine pyrophosphate, and 1.0 mM NAD^+^ to sonicated mitochondria (0.03 mg/mL protein).

### 4.5. Assay of ETC Complexes and ATP Hydrolase Activity

Activities of ETC CI-CIV were evaluated after exposure of cardiac mitochondria to various experimental conditions. CI activity was determined at 30 °C as the rate of NADH oxidation (340 nm, *ε* = 6.2 mM^−1^·cm^−1^) in assay buffer (35 mM KH_2_PO_4_, 5 mM MgCl_2_, 0.5 M EDTA and 2 mM KCN; pH 7.25) and upon the addition of 5 μM antimycin A, 60 μM decylubiquinone, and 0.1 mM NADH [54] to mitochondria (5 μg/mL protein). CII activity was assayed according to a method of Powell and Jackson [55]. Heart mitochondria (0.05 mg/mL) were placed into a solution consisting of 20 mM potassium phosphate buffer (pH 7.6), 30 mM succinate, 3 mM NaCN, and 1.33 mM phenazine methosulphate, and the reaction was initiated after the addition of 0.07 mM 2,6-dichloroindophenol at 37 °C (600 nm, *ε* = 21 mM^−1^·cm^−1^). For analysis of CIII, mitochondrial proteins (5 μg/mL) were suspended in buffer (35 mM KH_2_PO_4_, 5 mM MgCl_2_, and 2 mM KCN; pH 7.25) and disrupted with 0.05% Triton X-100. Enzyme activity was determined at 30 °C as the rate of 50 μM cytochrome c reduction at 550 nm (*ε* = 18.5 mM^−1^·cm^−1^) upon the final addition of 60 μM decylubiquinol to the reaction. CIV activity was measured as the rate of cytochrome c oxidation at 550 nm (*ε* = 19.1 mM^−1^·cm^−1^); 5 μg/mL mitochondrial proteins were incubated at 30 °C in medium containing 50 mM Tris-HCl (pH 8.0), 0.01% (*w*/*v*) n-dodecyl β-d-maltoside. The reaction was started by the addition of 5 μM reduced cytochrome c [53]. The oligomycin-sensitive activity of ATP hydrolase (CV) was determined by a coupled assay in which ADP production was linked to NADH oxidation and measured at 340 nm (30 °C, *ε* = 6.2 mM^−1^·cm^−1^) according to previously published methods [56,57]. Mitochondria (5 μg/mL) were added to the assay medium containing 250 mM sucrose, 50 mM KCl, 20 mM Tris-HCl adjusted to pH 7.4; 2 mM MgCl_2_, 2 mM phosphoenolpyruvate, 0.01 mg/mL lactate dehydrogenase, 0.02/mL pyruvate kinase, 0.02 mM rotenone, and 0.2 mM NADH. The reaction was initiated by the addition of 2.5 mM ATP.

### 4.6. Statistics and Bioinformatics

The data were explored and analyzed by R [(1); R Core Team (2018). R: A language and environment for statistical computing. R Foundation for Statistical Computing, Vienna, Austria. URL https://www.R-project.org/], ver. 3.5.2, with the aid of libraries beeswarm [(2); Aron Eklund (2016). beeswarm: The Bee Swarm Plot, an Alternative to Stripchart. R package version 0.2.3. https://CRAN.R-project.org/package=beeswarm], ROCit [(3); Md Riaz Ahmed Khan and Thomas Brandenburger (2019). ROCit: Performance Assessment of Binary Classifier with Visualization. R package version 1.1.1. https://CRAN.R-project.org/package=ROCit], randomForestSRC [(4); Ishwaran H. and Kogalur U.B. (2019). Fast Unified Random Forests for Survival, Regression, and Classification (RF-SRC), R package version 2.9.1.], ggRandomForests [(5); John Ehrlinger (2016). ggRandomForests: Visually Exploring Random Forests. R package version 2.0.1. https://CRAN.R-project.org/package=ggRandomForests]. A boxplot overlaid with swarmplot (2) was used for data visualization. A 2F-ANOVA with an interaction term was used to test the null hypothesis of the equality of population means among the factors, followed by the Tukey HSD *post-hoc* test with the adjusted *p*-values. Results with the *p*-value below 0.05 were considered statistically significant. The predictive power of diet or genotype was assessed by the empirical eROC curve (3) and quantified by the area under ROC. Uncertainty of the ROC curve estimate was quantified by the 95% confidence band. In addition to the above statistical analyses, the data were subjected to machine-learning predictive modeling by the random forest algorithm (4). Enzyme activities were used as predictors of either the *Slc41a1* genotype or the diet. Important predictors (enzymes) were identified by means of the nested cross-validation algorithm with the minimum graph depth criterion (4). The predictive performance of the resulting subset of selected important enzymes was visualized by the ROC curve (5), based on the out-of-bag data, thus providing a realistic estimate of the predictive performance of the algorithm on future data.

Where applicable, data are presented as a mean ± SEM; *p*-values below 0.05 were considered significant.

## 5. Conclusions

In summary, our findings demonstrate a prominent impact of dietary Mg on the functional capacity of key components of the energy-producing machinery in cardiac mitochondria. Dietary Mg depletion quenched the activity of Krebs cycle components (ACON, ICDH and KGDH), and consequently production of ETC substrates (reducing equivalents transporting molecules NAD^+^ and FAD). Our analyses also suggest a compensatory effect of functional SLC41A1 disruption on the effect of LMgD on activities of Krebs cycle enzymes ACON, ICDH and KGDH. Thus, for the first time, our data suggest a link between the presence of intact *Slc41a1*, thus the functionality of Na^+^/Mg^2+^ exchanger SLC41A1, and mitochondrial energy production. Furthermore, we hypothesize that insufficient supply of dietary Mg leads to decreased activities of particular ETC complexes in vivo, perhaps compensated by overexpression of these complexes aiming to sustain ETC efficiency. This assumption is supported by our findings in vitro where excessive amounts of Mg and substrates revealed increased activities of particular ETC complexes in mitochondria from Mg-deprived animals. Dysfunction of SLC41A1 had mostly similar effects on ETC as Mg starvation. The latter may point towards other functions of SLC41A1 in cellular physiology (e.g., in cellular signaling) that may prevail its function in Mg homeostasis in respect to ETC. It might also be possible that the activation of yet unknown transport mechanism, with similar *modus operandi* to SLC41A1 in respect to Mg^2+^ transport, substitutes SLC41A1 functionally.

Overall, our pivotal data further support that hypothesis that other Mg^2+^ transporting systems or Mg homeostatic factors of the cytoplasmic membrane (e.g., TRPM6/7, CNNM2, CNNM4) may equally impact on energy production in mitochondria. It remains to be tested whether the communication between SLC41A1 (or other Mg transporters/homeostatic factors) and the mitochondrial energy-producing machinery is simply dependent on their effects on cytoplasmic [Mg^2+^] and matrix [Mg^2+^], or whether a more intricate network of various signaling cascades is involved.

Magnesium is increasingly shown to play important roles in the molecular physiology of the cardiovascular system. Our work clearly indicates that a longer-lasting lack of dietary Mg and/or a lack of the functional Na^+^/Mg^2+^ exchanger SLC41A1 influence Krebs cycle and ETC and thus modulate mitochondrial energy production in heart mitochondria and probably global heart physiology. The effect of *Slc41a1* knock-out on integrative heart physiology should now be particular subject of further studies.

## Figures and Tables

**Table 1:** Brief data summary.

gnt.	Diet	Comparison	ACON	ICDH	KGDH	CI	CII	CIII	CIV	CV
bal.		(LMgD/NMgD)	↓	↓	↓	↑	–	–	↑	↑
	bal.	(*A1*^−/−^/*A1*^+/+^)	↑	ξ↑	–	↑	↓	↑	↑	↑
*A1* genotype × Mg diet			NS	NS	⦿	NS	NS	NS	***	*
*A1* ^−/−^		(LMgD/NMgD)	⦿↓	↓	↓	↑	–	–	↑	↑
*A1* ^+/+^		(LMgD/NMgD)	–	↓	–	↑	–	–	⦿↑	↑
	LMgD	(*A1*^−/−^/*A1*^+/+^)	–	–	–	–	⦿↓	↑	↑	↑
	NMgD	(*A1*^−/−^/*A1*^+/+^)	↑	–	–	–	↓	↑	↓	–

Symbols and abbreviations: –, no change; ↓, decreased activity; ↑, increased activity; ξ, nearly significant (*p* < 0.06); ⦿, trend towards significance (*p* < 0.1); *, *p* < 0.05; ***, *p* < 0.001; *A1*, *Slc41a1*; ACON, aconitate hydratase; bal., balanced for; CI, complex I; CII, complex II; CIII, complex III; CIV, complex IV; CV, complex V/F_1_F_o_-ATPase; gnt., genotype; ICDH, isocitrate dehydrogenase; int., interaction; KGDH, α-ketoglutarate dehydrogenase; LMgD, low Mg diet; NMgD, normal Mg diet.

## Abbreviations

2F-ANOVA	two-factorial analysis of variance
ACON	aconitate hydratase
Akt/PKB	Akt protein kinase B
AUC	area under the curve
CI–CV	complex I–V
CVD	cardiovascular diseases
CytC	cytochrome C
ECS	extracellular space
EGF	epidermal growth factor
eROC	empirical receiver operating characteristic
ETC	electron transport chain
Flp	flippase
FRT	flippase recognition target
HEK293	human embryonic kidney 293 cells
ICDH	isocitrate dehydrogenase
ICS	intracellular space
IGF-1	insulin-like growth factor 1
IR	insulin receptor
KGDH	α-ketoglutarate dehydrogenase
KO	knock-out
LMgD	low Mg diet
*loxP*	locus of X-over P1 sites
[Mg^2+^]_e_	extracellular Mg^2+^ concentration
[Mg^2+^]_i_	intracellular/cytosolic Mg^2+^ concentration
[Mg^2+^]_m_	mitochondrial/matrix Mg^2+^ concentration
MIMS	mitochondrial intermembrane space
NME	Na^+^/Mg^2+^ exchanger
NMgD	normal Mg diet
PDGF	platelet-derived growth factor
PDH	pyruvate dehydrogenase
PDK1	3-phosphoinositide-dependent protein kinase 1
PI3K	phosphatidylinositol 3-kinase
PKA	protein kinase A
Q	coenzyme Q10
RFM-L	random forest machine-learning
ROC	receiver operating characteristic
*Slc41a1*/SLC41A1	solute carrier family 41 member A1 (*gene*/protein)
TCA	tricarboxylic acid cycle
TRPM7	transient receptor potential melastatin type 7
WT	wild-type
